# Comprehensive Outcomes of the Keystone Design Perforator Island Flap: A Prospective Study of 121 Consecutive Cases

**DOI:** 10.1055/a-2668-4004

**Published:** 2025-11-20

**Authors:** Sofija Pejkova, Gordana Georgieva, Sofija Tusheva, Katerina Jovanovska, Stefania Azmanova Mladenovska, Bisera Nikolovska, Blagoja Srbov

**Affiliations:** 1 University Clinic for Plastic and Reconstructive Surgery, Faculty of Medicine, University Ss. Cyril and Methodius, Skopje, North Macedonia

**Keywords:** Keystone flap, reconstruction, KDPIF, perforator flap, sensibility

## Abstract

**Background:**

The Keystone Design Perforator Island Flap (KDPIF), developed by Behan in 2003, has significantly impacted reconstructive surgery. This technique utilizes angiotomes, which integrate vascular, dermatomal, neural, and lymphatic networks, providing various approaches for complex reconstructions.

**Methods:**

This study enhances the initial PACE framework (Pain, Aesthetic appearance, Complication rates, Economic feasibility) by adding “S” for Sensibility, creating the PACES framework to include sensory improvements in the assessment.

Our retrospective analysis included 121 patients over 51 months, primarily with defects from skin malignancies (66.9%) and chronic wounds (16.5%), mainly on the trunk (51.2%). The average age was 56.2 years, with males comprising 73.6% of the cohort.

**Results:**

Outcome measures included pain, assessed using the Visual Analog Scale at 1 and 12 months postoperatively, revealing a significant reduction in pain. Aesthetic outcomes were evaluated through the Manchester Scar Scale, showing notable improvements in scar appearance. Complication rates were low, indicating the safety of the flap in this series. Economic efficiency was assessed by operative time and hospital stay, with shorter durations indicating cost-effectiveness.

Sensory outcomes, measured with the Postoperative Flap Sensitivity Self-Assessment Questionnaire, showed substantial gains in sensory function, enhancing patients' quality of life.

**Conclusion:**

The PACES framework facilitated a comprehensive evaluation of KDPIF, confirming its effectiveness across various defect types and patient populations. Our findings support the wider adoption of KDPIFs for reconstructive purposes, alongside ongoing efforts to optimize patient outcomes and functionality.

## Introduction

In 2003, the Keystone Design Perforator Island Flap (KDPIF) was first described by Behan and has been widely recognized as a significant advancement in reconstructive surgery. In his initial publication, Behan delineated the unique design of KDPIF, explained as a flap based on angiotomes combining vessels and dermatomes, and for supplementary neural and lymphatic input. This innovative technique has shown great results in different patient populations such as those with trauma, cancer resections, or persistent non-healing wounds. The concept of this study is to analyze the KDPIF from different perspectives using the PACES framework: Pain evaluation, Aesthetic appearance evaluation, Complication rates monitoring, Economic feasibility assessment, and Sensory examination. 1

This research sets out to comprehensively evaluate KDPIFs so that reconstructive surgeons can make decisions based on evidence about their use. The aim of this investigation is to assess the wide applicability of these flaps not only for better surgical outcomes but also to understand more about what they achieve beyond technical success.

## Methods

### Study Design

In this study, we performed a prospective analysis of 121 patients' data who had reconstructive surgery with the KDPIF over 51 months in our hospital. The study was approved by the Institutional Review Board. All patients provided written informed consent for participation in the study, including permission for the use of anonymized clinical photographs in scientific publications.

### Data Collection

We compiled a comprehensive list of variables. Regarding the patients' demographics, we kept detailed records on age, sex, and underlying diseases. Characteristics of the defects were also recorded in detail, such as traumatic etiology, oncologic cause, or due to chronic wounds, among others; location and size were some anatomical features considered too, which are displayed in Table 1 .

**Table 1:** Detailed characteristics of the patients, including defect etiology, anatomical location, size, and hospital stay

Patient ID	Gender	Age	Defect site	Defect dimensions	Semimajor axis a (cm)	Semiminor axis b (cm)	Defect area (cm ^2^ )	Hospital days
0001/18	F	64	Dorsal	M	4.79	3.2	48.11	3
0001/19	F	46	Dorsal	L	6.07	4.05	77.28	3
0001/20	M	72	Dorsal	M	4.68	3.12	45.8	2
0001/21	M	42	Hand	M	4.63	3.08	44.85	7
0001/22	M	28	Sacral	L	6.1	4.07	78.04	5
0002/18	F	60	Dorsal	M	5.09	3.39	54.18	3
0002/19	M	58	Knee	S	3.26	2.17	22.21	2
0002/20	M	83	Hand	S	3.1	2.06	20.08	1
0002/21	M	70	Shoulder	M	5.09	3.39	54.16	5
0002/22	M	25	Sacral	L	6.03	4.02	76.17	5
0003/18	F	65	Dorsal	M	4.98	3.32	51.88	2
0003/19	F	81	Dorsal	M	4.91	3.27	50.47	3
0003/20	M	58	Pectoral	S	3.1	2.06	20.08	0
0003/21	M	79	Auricular	S	3.57	2.38	26.67	5
0003/22	M	24	Sacral	L	6.2	4.13	80.44	4
0004/19	F	87	Sternal	S	3.64	2.43	27.82	2
0004/20	M	67	Crural	M	4.62	3.08	44.77	0
0004/21	F	35	Sacral	M	4.75	3.17	47.23	2
0004/22	M	31	Axillar	M	4.68	3.12	45.8	2
0005/19	M	70	Dorsal	M	4.68	3.12	45.8	2
0005/20	M	51	Dorsal	S	3.02	2.01	19.13	0
0005/21	M	52	Sacral	M	4.92	3.28	50.61	5
0005/22	M	58	Hand	S	3.08	2.06	19.92	1
0006/19	M	64	Face	S	3.5	2.33	25.69	2
0006/20	M	72	Femoral	M	5.04	3.36	53.29	3
0006/21	F	49	Inguinal	M	4.67	3.11	45.63	2
0006/22	M	20	Sacral	L	5.95	3.97	74.16	4
0007/19	F	41	Brachial	S	3.41	2.28	24.39	3
0007/20	M	63	Dorsal	M	4.91	3.27	50.5	2
0007/21	M	25	Femoral	M	5.1	3.4	54.38	1
0007/22	M	76	Face	S	3.2	2.13	21.41	2
0008/19	M	28	Femoral	L	6.37	4.25	84.94	3
0008/20	M	73	Dorsal	M	4.96	3.31	51.62	2
0008/21	F	73	Crural	S	3.14	2.09	20.63	0
0008/22	M	26	Sacral	L	6.03	4.02	76.19	4
0009/20	M	72	Head	S	3.59	2.39	27	0
0009/21	M	76	Face	S	3.12	2.08	20.33	2
0009/22	M	29	Axillar	L	6.07	4.05	77.17	2
0010/20	M	36	Abdominal	M	4.6	3.07	44.38	0
0010/21	F	66	Face	S	3.12	2.08	20.35	0
0010/22	M	17	Sacral	L	6.12	4.08	78.37	4
0011/20	M	76	Dorsal	M	5.1	3.4	54.38	1
0011/21	M	70	Dorsal	M	4.75	3.17	47.24	0
0011/22	M	50	Sacral	L	6.29	4.19	82.74	4
0012/20	M	80	Dorsal	M	5.03	3.35	52.93	2
0012/21	F	36	Dorsal	M	4.79	3.19	48.02	3
0012/22	M	45	Crural	M	4.68	3.12	45.8	1
0013/20	M	70	Shoulder	M	4.71	3.14	46.4	0
0013/21	M	66	Dorsal	M	4.84	3.22	48.97	3
0013/22	M	21	Sacral	L	5.98	3.99	74.96	1
0014/20	M	70	Crural	M	4.69	3.13	46.08	6
0014/21	M	58	Crural	S	3.21	2.14	21.52	21
0014/22	M	63	Sacral	L	6.15	4.1	79.14	8
0015/20	F	66	Crural	M	4.69	3.13	46.09	30
0015/21	F	56	Gluteal	S	3.36	2.24	23.67	4
0015/22	M	35	Neck	L	6.19	4.12	80.18	1
0016/20	M	37	Scrotal	M	4.76	3.17	47.37	6
0016/21	M	71	Pectoral	M	4.7	3.13	46.26	3
0016/22	M	31	Axillar	M	4.62	3.08	44.77	2
0017/20	M	70	Sternal	S	3.41	2.28	24.41	0
0017/21	M	72	Face	M	5	3.34	52.44	2
0017/22	M	45	Foot	M	5.04	3.36	53.29	3
0018/20	M	67	Shoulder	M	4.87	3.25	49.69	0
0018/21	M	43	Sternal	M	4.7	3.13	46.26	2
0018/22	M	33	Sacral	M	4.91	3.27	50.5	6
0019/20	M	69	Pectoral	M	4.82	3.22	48.71	0
0019/21	M	76	Dorsal	L	6.19	4.13	80.29	5
0019/22	M	70	Abdominal	L	5.9	3.93	72.92	3
0020/20	F	71	Antebrachial	S	3.49	2.32	25.46	0
0020/21	M	68	Dorsal	L	6.25	4.16	81.72	10
0020/22	F	73	Dorsal	L	6.2	4.13	80.38	9
0021/20	M	73	Pectoral	L	6.26	4.17	82.04	5
0021/21	M	34	Brachial	M	4.87	3.24	49.58	3
0021/22	M	76	Dorsal	L	5.97	3.98	74.57	3
0022/20	M	77	Foot	L	6.37	4.25	84.92	1
0022/21	F	71	Antebrachial	M	4.91	3.27	50.4	4
0022/22	M	42	Sacral	L	5.91	3.94	73.17	5
0023/20	M	55	Femoral	M	4.75	3.17	47.23	0
0023/21	F	46	Crural	M	4.62	3.08	44.65	2
0023/22	M	77	Shoulder	M	4.96	3.31	51.62	2
0024/20	F	65	Antebrachial	M	4.92	3.28	50.61	4
0024/21	F	59	Scapular	L	5.89	3.92	72.57	10
0024/22	M	67	Brachial	S	3.25	2.17	22.13	2
0025/20	M	85	Dorsal	L	6.19	4.13	80.26	14
0025/21	F	62	Shoulder	L	6.38	4.25	85.2	6
0025/22	M	84	Auricular	S	3.31	2.21	23	7
0026/20	F	52	Inguinal	L	5.96	3.97	74.38	3
0026/21	M	34	Head	L	6.31	4.21	83.37	7
0026/22	F	70	Antebrachial	M	4.6	3.07	44.38	7
0027/20	M	60	Hand	S	2.99	2	18.76	4
0027/21	M	57	Neck	L	5.99	3.99	75.12	1
0028/20	M	37	Inguinal	L	5.96	3.97	74.37	4
0028/21	M	26	Sacral	M	4.91	3.28	50.56	7
0029/20	M	32	Hand	S	3.66	2.44	28.01	1
0029/21	M	49	Hand	S	3.3	2.2	22.77	4
0030/20	M	83	Pectoral	M	4.67	3.11	45.63	1
0030/21	M	54	Crural	M	4.68	3.12	45.96	7
0031/20	M	52	Neck	M	4.75	3.17	47.24	3
0031/21	M	85	Cubital	L	5.97	3.98	74.72	1
0032/20	M	22	Sacral	M	4.79	3.19	48.02	4
0032/21	M	21	Sacral	M	4.63	3.08	44.85	7
0033/20	M	63	Sacral	L	5.91	3.94	73.07	6
0033/21	M	70	Face	S	3.2	2.13	21.4	9
0034/20	M	57	Knee	M	4.84	3.22	48.97	16
0034/21	M	64	Face	S	3.42	2.28	24.52	4
0035/20	M	32	Sacral	M	5	3.34	52.44	7
0035/21	F	50	Dorsal	M	5.09	3.39	54.16	2
0036/20	M	74	Pectoral	L	6.33	4.22	83.82	9
0036/21	M	68	Foot	M	4.79	3.2	48.11	2
0037/20	M	88	Scapular	M	4.7	3.13	46.26	0
0037/21	M	21	Sacral	M	5.09	3.39	54.18	3
0038/20	F	78	Antebrachial	M	4.87	3.24	49.58	9
0038/21	F	68	Gluteal	L	5.97	3.98	74.74	14
0039/20	M	18	Sacral	M	4.91	3.27	50.4	4
0039/21	M	58	Dorsal	M	4.98	3.32	51.88	2
0040/20	F	60	Crural	M	4.62	3.08	44.65	8
0040/21	F	67	Knee	L	6.04	4.03	76.35	13
0041/20	F	50	Femoral	M	4.91	3.28	50.56	6
0041/21	M	70	Sacral	M	4.91	3.27	50.47	5
0042/20	M	19	Sacral	M	4.68	3.12	45.96	4
0042/21	M	69	Sacral	L	6.15	4.1	79.28	70

Abbreviations: F, female; M, male; S, small; M, medium; L, large.

### Keystone Design Perforator Island Flap Design

The surgical lesion should be excised in an elliptical shape, aligning its axis with the natural course of cutaneous nerves, veins, or known vascular perforators. In the upper and lower limbs, this orientation is typically longitudinal. After the lesion is removed, the KDPIF is designed with a 1:1 ratio between the width of the defect and the width of the flap. The flap's length is determined by the size of the excised defect. At the edges of the excision, right angles are created to form the keystone configuration.

Blunt dissection enables the mobilization of surrounding tissue, allowing the flap to advance and aiding in wound closure. The initial step in closure involves direct approximation of the defect using interrupted single-layer nylon sutures. Depending on the defect's size, two to four stay sutures may be required in this closure technique. Next, a V–Y advancement is performed at both ends of the flap along the longitudinal axis. This maneuver creates redundancy and laxity at the right-angle points of the flap, which are subsequently excised, further narrowing the defect.

The final closure of the relaxed keystone flap is achieved using a hemming suture, advancing it horizontally into the original defect before securing it. Additional undermining and tissue release help distribute tension evenly, facilitating circumferential wound closure. A continuous everting horizontal mattress suture can further aid in evenly dispersing tension around the flap margins.

Closing the V–Y points initially may help reduce tension. However, our approach prioritizes direct closure at the midpoint, allowing for an early assessment of tension levels. To prevent wound dehiscence, stay sutures should remain intact for 14 to 17 days.

#### Classification of Keystone Design Perforator Island Flap

**Fig. 1:**
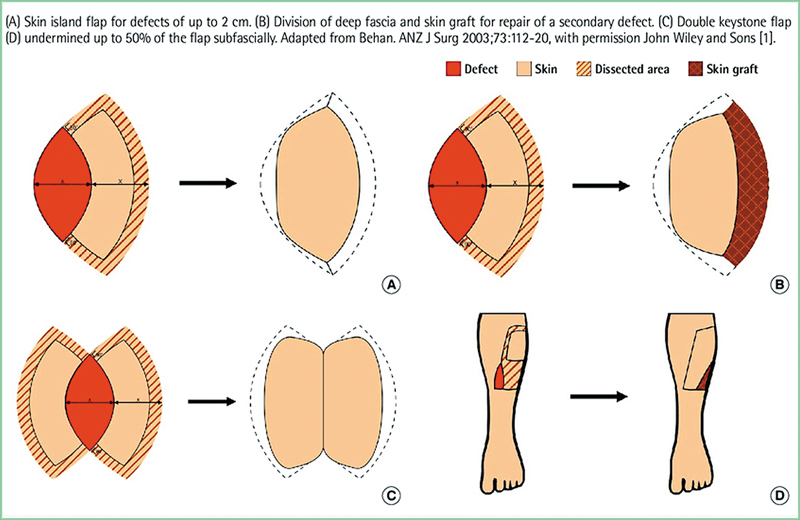
Traditional classification of the keystone flap. A systematic review of the keystone design perforator island flap in the reconstruction of trunk defects: Scientific Figure on ResearchGate. Available from: https://www.researchgate.net/figure/Traditional-classification-of-the-keystone-flap_fig1_347073665 (accessed February 6, 2025).

KDPIF are classified as Type I, Type II, Type III, and Type IV, as well as Omega modification, which have various perforator patterns as well as angiotome distributions. 2 3 4 The use of this classification system allows for personalized design to each defect at different surgical sites ( Fig. 1 ).

**Fig. 2:**
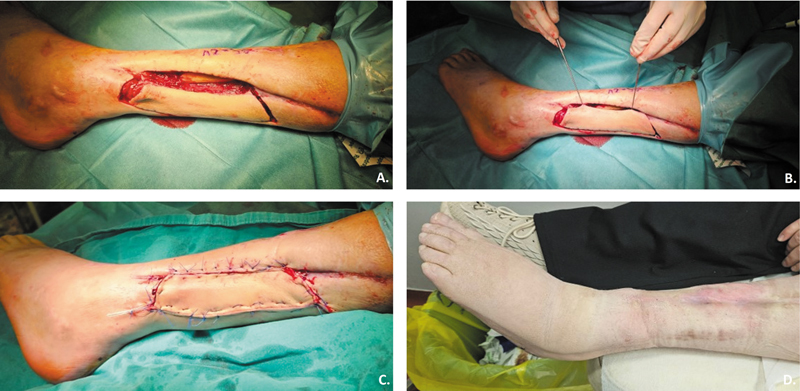
Type II Keystone Design Perforator Island Flap for lower limb defect reconstruction.

**Fig. 3:**
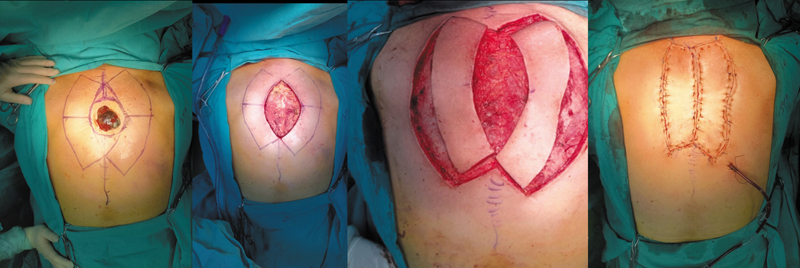
Type III Keystone Design Perforator Island Flap for back defect reconstruction.

Type I KDPIF utilizes one perforator, where small defects need blood supply from flaps that should be repaired, while type two depends on several perforators supporting larger flaps, thereby providing flexibility when dealing with moderately sized defects during reconstruction surgery. 5 On the other hand, type three involves utilization of a multiple angiotome approach towards managing bigger complex defects. 1 6 7 8 Finally, type IV integrates neighboring angiotomes so that viability can still be assured even if the primary angiotome is compromised ( Figs. 2 and 3 ). 9

**Fig. 4:**
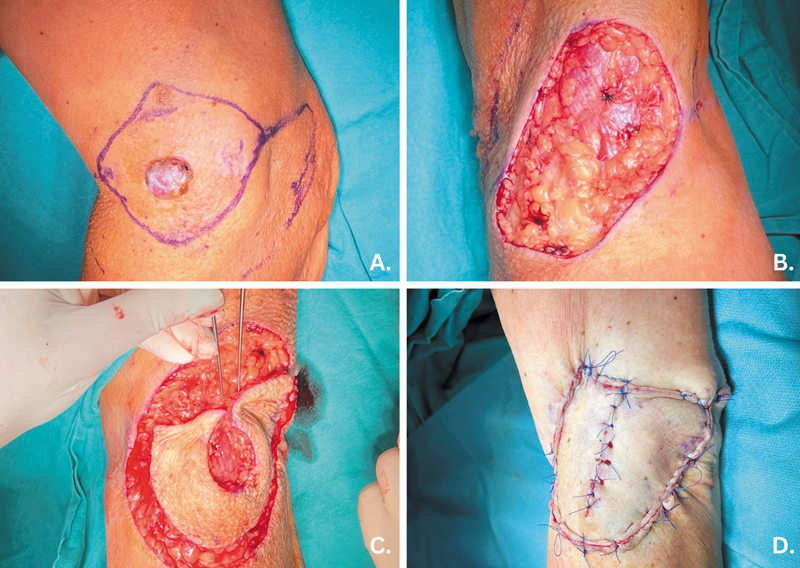
Omega modification Keystone Design Perforator Island Flap used for complex defect sites.

Omega modification increases versatility by adding extra branches or extensions into consideration during design optimization of KDPIFs for complex defect sites ( Fig. 4 ). 10

### Outcome Measures

The pain was assessed quantitatively through the Visual Analog Scale (VAS) during follow-ups at first and twelfth months after the surgery. This approach can be used to measure the intensity of pain both in short-term and long-term recovery periods. 11

#### Aesthetic Acceptability

The Manchester Scar Scale (MSS) was employed to evaluate the aesthetic qualities of scars at 1- and 12-month postoperative visits. This scale examines different features like color, surface texture, distortion, border irregularity, vascularity, or pigmentation. 12 Each feature is scored separately with an overall score from 4 to 14 points, with higher scores implying poorer cosmetic outcome. 13

#### Complications

During the early postoperative phase, attention was focused on immediate complications such as infections, skin necroses, and wound dehiscence.

#### Economic Efficiency

Economic efficiency was measured using operating time and length of hospital stay at first discharge or any subsequent readmission within a year after operation. These values are crucial in determining cost-effectiveness related to various surgeries since they reveal resource utilization and impact upon the health care system. 14

#### Sensory Assessment

**Fig. 5:**
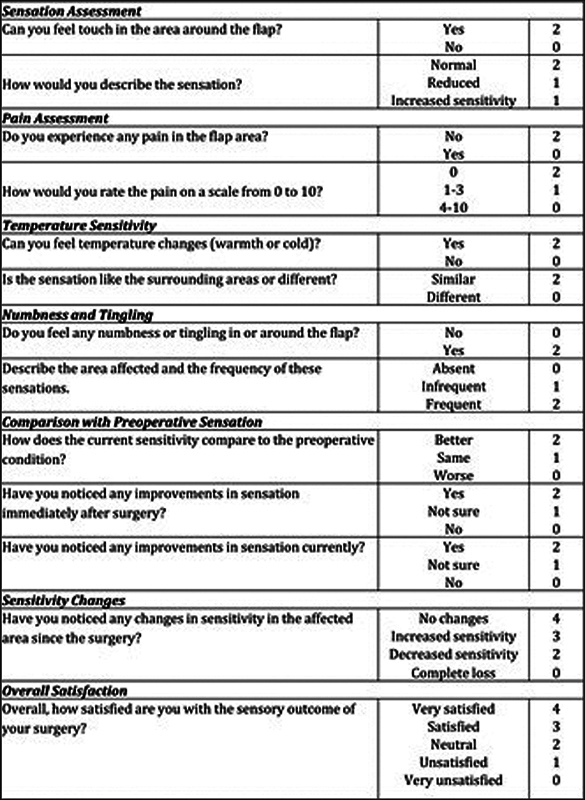
The maximal score equals 30 points. Points between 24 and 30 mean excellent recovery with high satisfaction; between 18 and 23 good recovery with manageable issues; between 12 and 17 fair recovery with noticeable problems affecting quality of life; between 6 and 11 poor recovery associated with significant sensory dysfunction; between 0 and 5 very poor outcome due to major deficits causing functional disability.

Sensory outcomes following KDPIF reconstruction were evaluated using subjective measurements through the “Postoperative Flap Sensitivity Self-Assessment Questionnaire” patient-reported outcome measures (PROMs). In this study, patients' flap sensitivity 1 year postoperatively was assessed with a questionnaire consisting of several sections that evaluated pain, touch sensation, overall satisfaction with the surgical result, numbness/tingling, and temperature sensitivity. Each section had its own scoring system depending on patients' self-reported experience ( Fig. 5 ).

### Data Analysis

All data were analyzed according to the PACES acronym framework (Pain, Aesthetic acceptability, Complication rates, Economic effectiveness, Sensibility), which allows for an extensive evaluation of KDPIF across these essential factors, thereby giving a wider understanding of its applicability.

### Statistical Analysis

Descriptive statistics were used to summarize the study population and defects reconstructed with KDPIF using IBM SPSS 24 statistical software package.

## Results

Out of 121 patients in the cohort, 73.6% were male, and 26.4%. Participants' mean age was 56.2 years (range 17–78 years).

Soft tissue defects after skin cancer excisions were 66.9%. Posttraumatic defects represented 7.4%, chronic wounds accounted for 16.5% and pilonidal cyst excisions defects that required repair were 9.1%. The majority (51.2%) of these reconstructions were done on the trunk; other areas involved were lower limb (19.8%), upper limb (16.5%), and head/neck region (12.4%). This means that more KDPIFs are used on the trunk than any other part because it has a larger surface area with complex defect presentations common to this region.

Type II flaps were most frequent in 52.1%. This was followed by Type I at 20.7%, Type III at 9.9%, and only Type IV at 1.7%. Omega modification was employed in 15.7% of patients, suggesting a diverse approach to flap selection based on specific patient needs and defect characteristics. Preference for type II flaps could be attributed to their versatility since they can address various reconstructive challenges effectively.

### Pain

Pain was assessed using VAS at 1 and 12 months postreconstruction to cover both immediate and later outcomes.

**Fig. 6:**
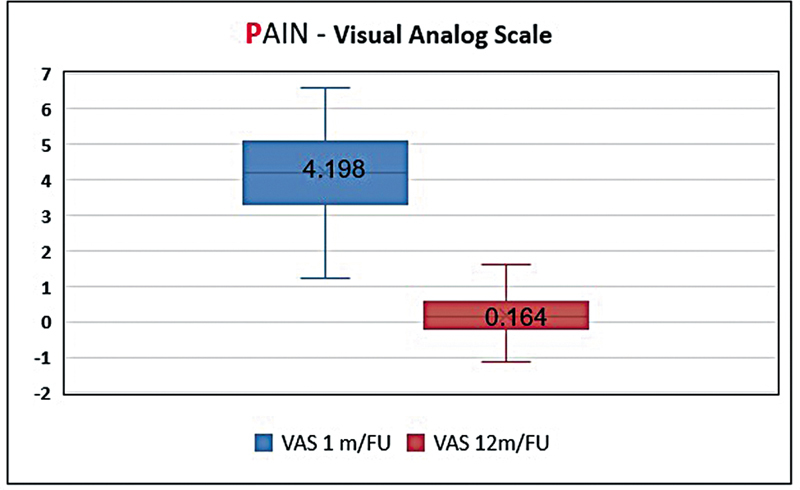
The Visual Analog Scale (VAS) score for pain 1 month postoperatively showed a significant decrease by the 12-month mark.

At 1-month follow-up, initial results provided baseline data on early postoperative outcomes, while 12-month assessments gave insights into long-term pain management. On evaluation, the mean VAS score for pain 1 month postoperatively showed a value of 4.198, significantly dropping to 0.164 at 12 months ( *p* < 0.001; Fig. 6 ).

These findings are supportive of the PACES framework—Pain, Aesthetic outcomes, Complications, Economic costs, Sensory improvement—for evaluating KDPIF reconstructions' outcomes regarding the Pain component.

### Aesthetics

Scars were evaluated using the MSS at two time points—1 and 12 months postreconstruction—selected to represent immediate and long-term effects of reconstruction of defects with KDPIF.

Data from the 1-month follow-up established a baseline for early outcome, while the 12-month findings gave an indication of late-stage stability and quality of scar maturation. A comparison between these periods demonstrated statistically significant improvements ( *p* < 0.05) in aesthetics at 12-month follow-up.

**Fig. 7:**
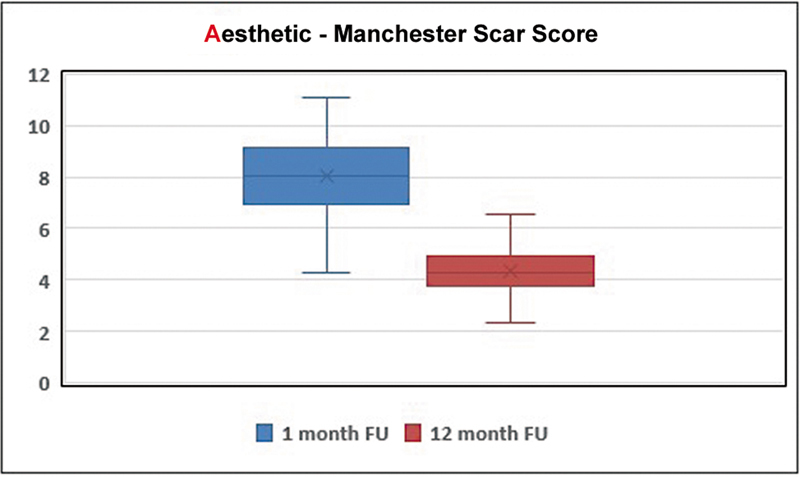
A comparison between these periods revealed statistically significant improvements ( *p* < 0.05) in aesthetic outcomes at the 12-month follow-up.

According to our results, the MSS has provided a strong method for assessing objective aesthetic features following KDPIF reconstruction. The results demonstrate improvement over time, thus supporting its use within broader PACES outcome measures ( Fig. 7 ).

### Complications

Hematoma, infection, partial skin necrosis, and dehiscence were specific postoperative complications noted. It is estimated that about 13.1% of them had a hematoma, which was the most common complication. Infections occurred in 2.5% of people, a rate much lower than other reconstructive surgeries, where it can be up to seven times more frequent. Partial skin necrosis occurred in 7.4% while dehiscence appeared in 3.3% patients treated by this method. All these complications were noted in the early postoperative period and resolved with local treatment without compromising flap survival.

**Fig. 8:**
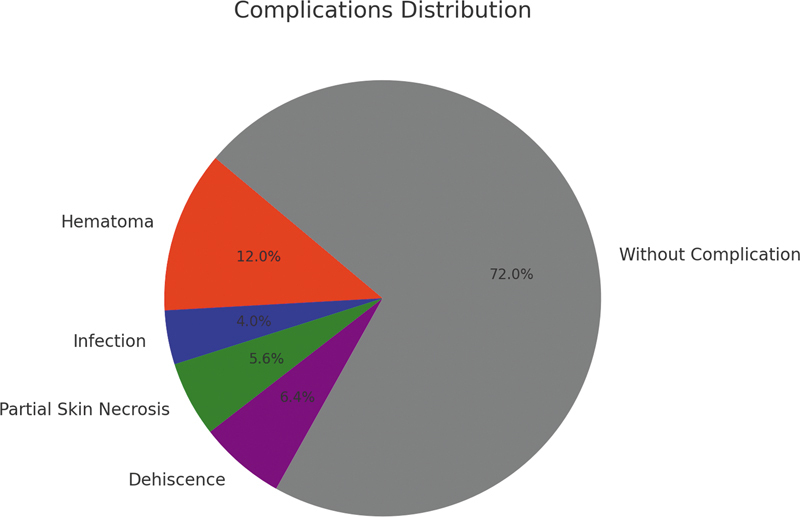
Positive results indicated that 72% of patients experienced no complications after surgery, suggesting the inherent safety of Keystone Design Perforator Island Flap. This finding supports its efficacy in providing sufficient vascular supply and the flexibility needed for successful tissue integration and healing.

Positive results also showed that 72% patients had no complication at all after surgery, suggesting the safety inherent to KDPIF but also supporting its efficacy in providing enough vascular supply as well as flexibility required for successful tissue integration and healing ( Fig. 8 ).

### Economic

Two parameters were considered: The duration of surgery and the length of postoperative hospital stay, both known to have significant cost implications.

More than half (53.7%) of all operations were completed within a relatively short span—between 30 minutes and 1 hour.

Also, from our data, we saw that most patients only stayed in the hospital for 1 to 5 days after their operation was done. We used analysis of variance (ANOVA) on our cohort to test if there is a relationship between how long surgeries took and the lengths of hospital stays among the patients we have. The results showed that there was significant variation among groups based on the times taken for different categories of surgeries. The surgeries were categorized into three: 0 to 30, 30 to 60, and 60 to 90 minutes.

Our findings show statistically that there are significant differences in durations spent at hospitals according to surgery duration ( *p* < 0.05). However, post hoc tests indicated that at least surgeries with shorter operative time should be selected since they lead to faster recovery and lower use of health care resources during the first 5 days following such operations than higher level ones ( *p* < 0.05). It suggests that middle-level surgeries are normally better when it comes to faster recovery and reduced use of health care resources.

**Fig. 9:**
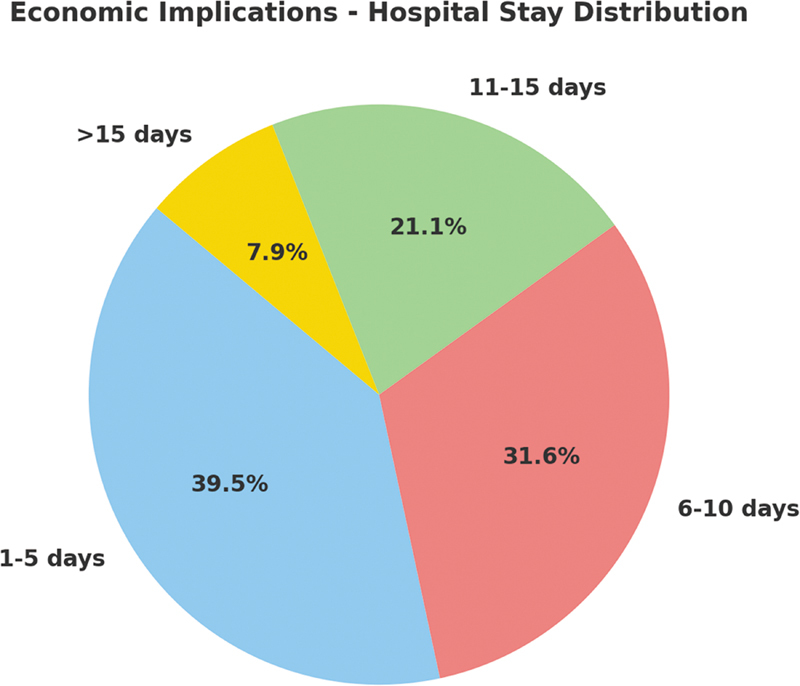
Economic implications of hospital stay distribution among patients undergoing keystone design perforator island flap (KDPIF) reconstruction. The majority of patients (39.5%) were discharged within 1-5 days, followed by 6-10 days (31.6%), 11-15 days (21.1%), and more than 15 days (7.9%). Shorter hospital stays highlight the cost-effectiveness of the procedure.

Furthermore, it is important to note that defect size determines how long a surgery will take because larger defects require more extensive reconstructions, which may prolong this period ( Fig. 9 ).

**Fig. 10:**
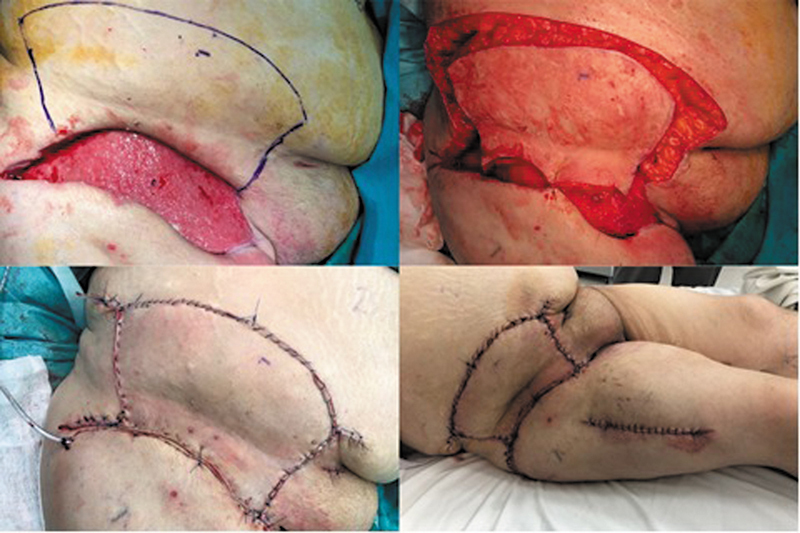
Keystone Design Perforator Island Flap was utilized for an inguinal defect in a patient with comorbidities.

To identify which subgroup of patients had the longest length of stay at the hospital, we conducted an ANOVA test on a group of 121 patients who underwent KDPIF reconstruction for soft tissue defects. These patients were divided into four categories depending on their comorbidities: Diabetes mellitus, peripheral arterial disease (PAD), cardiomyopathy, and a control group without any comorbidity ( Fig. 10 ).

According to the results from ANOVA, there is a significant effect of PAD on the duration of hospitalization. This means that through statistical analysis, there was a significant difference in lengths of stay among groups ( *p* < 0.001). It shows that those people who have PAD take much longer times in hospitals than other categories, hence, indicating its stronghold on hospitalization duration.

### Sensation

To evaluate the sensory restoration following KDPIF reconstruction, we used the “Postoperative Flap Sensitivity Self-Assessment Questionnaire” with PROMs to give insight into sensory recovery as seen by patients. This questionnaire evaluates various aspects of sensation, which are pain, touch, satisfaction with operation outcome, numbness and tingling, as well as temperature. A total of 121 patients were followed up for 1 year, and their senses were tested using this tool.

**Fig. 11:**
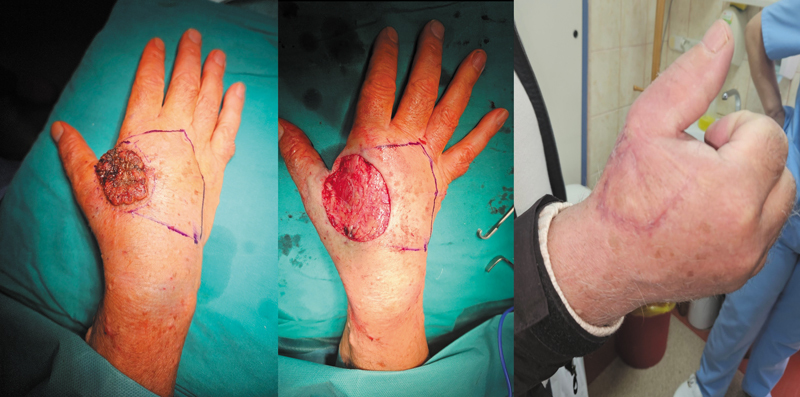
This progression suggests a surgical approach involving excision of a skin cancer on the hand followed by Keystone Design Perforator Island Flap reconstruction to restore hand function and aesthetics. The final image suggests good healing, with scarring limited to the area around the lesion.

ANOVA was done to check if there is any difference in sensory outcomes between different types of KDPIFs, such as I to IV, and Ω modifications. In terms of reconstruction method, five groups were created, based on which statistical significance was assessed using each group's sensory score ( Fig. 11 ).

## Discussion

Many reports have been published on the application and effectiveness of KDPIF in reconstructive surgery. However, there is a paucity of comprehensive studies that have investigated its use across various patient demographics and types of deformities. It has become increasingly apparent that as we increase our knowledge about KDPIFs, it would be necessary for us to improve their evaluation accordingly. We employed the PACES framework, from which we thought it wise to come up with a systematic methodology for determining outcomes, whereby the PACES were considered.

Behan's groundbreaking research revealed that KDPIFs could be used as an option for reconstruction of complex postexcisional defects. 8 These findings are comparable with ours as they are applicable to different demographic profiles and defects. 15 16 17 This study showed how practical KDPIFs can be in various clinical settings by involving patients who belong to different demographic groups. 18 19 20 21 22

The most common type of defect found was skin cancers located mainly on the trunk region. The results provided here support Behan's conclusion that the technique can be applied to many clinical scenarios, including those affecting skin cancers. 23 24 25 Also based on Behan's categorization model, Type IIa KDPIF was predominantly used due to its reliability in the reconstruction of a wide range of defects.

Our data provide conclusive proof that KDPIF is highly effective and versatile in reconstructive surgery, leading to positive outcomes for all components incorporated into the PACES frame. The results from our study may shape future inquiries that will necessitate alteration and augmentation of the procedure as well as its applications.

Pain constitutes one important measure for assessing success after surgery; it was assessed through VAS. 11 The reduction in VAS scores from 1-month postoperation and 12 months after the surgery (4.06–0.19) demonstrates that there was effectiveness of KDPIF in managing pain, and this is consistent with findings from other researchers. In clinical terms, our study's findings are important because they suggest that KDPFs can greatly enhance patient satisfaction and comfort following the surgery. As such, for clinicians, this means a possible decrease in long-term use of analgesics, hence ensuring patients' well-being while minimizing drug side effects. The substantial reduction in pain demonstrated by the VAS scores strongly supports the use of KDPIF for reconstructive purposes. The improvement from moderate to mild pain at 1-year follow-up confirms not only its effectiveness as a reconstructive method but also its appeal as a procedure that offers improved long-term control over pain and overall outcomes for patients.

The results of our study show that the use of “A” (for aesthetics) in PACES, an acronym for assessing KDPIF outcomes, is supported. This was clearly pointed out by results from MSS. Four primary visual attributes are measured by MSS on scars, that is, color, contour, distortion, and gloss. The lower scores will result in more desirable outcomes because this assessment provides a comprehensive measurement gauge to scar aesthetics. This was confirmed by a decrease in the MSS from 7.8 at 1 month to 4.4 at 12 months after the operation. Accordingly, KDPIF is very successful, and there has been supportive evidence proving it with a favorable improvement in scar appearance over time as well. The significance of considering aesthetic factors in patient care and treatment outcome evaluation is further emphasized.

These rates of early postoperative complications observed, related to 22.3% are within ranges defined by other investigators. 16 19 20 According to Behan et al., one of their most recurrent complications was hematoma which had occurred in 13.2% of patients and was managed in the early postoperative period. 13

The success found among this group matches up with “C,” which stands for complications, thus meaning that favorable surgical outcomes, and can be obtained if the selection of the type of reconstructive option is adjusted to different anatomical sites or patient-specific aspects during reconstructive operations. Each case's requirements may differ significantly; therefore, it means it is flexible enough. Therefore, greater use should be made of KDPIF across clinical settings, with different regions, so as to reduce severe morbidity rates and promote faster recovery.

Combining the length of stay in hospital with the operation time, we can obtain important indicators about a patient's recovery after surgery. Also, these data indirectly tell us about the economic implications of using KDPIF. Shorter stays in hospitals indicate quicker recoveries, less resource use, and potential savings in costs. Furthermore, this indicator reflects the impacts generated by efficiency on different health care systems.

These insights are important in surgical practice because they explain how KDPIF can improve clinical outcomes and economic resources. In relation to cost containment, even patients undergoing major and complex reconstructions may be discharged early. This technique provides low-cost surgeries, while at the same time ensuring patient-centeredness and facilitating fast healing. It is highly efficient for both surgeons and patients as it conjoins surgical accuracy with economic and pragmatic health needs.

The results of our study for sensory recovery were evaluated with the Postoperative Flap Sensitivity Self-Assessment Questionnaire with PROMs to give insight into sensory recovery as seen by patients. They showed high sensory recovery rates, whereby 86.8% of cases reported improvement over the 12-month follow-up period attributable to KDPIFs. Different types of KDPIF reconstruction influence sensory recovery differently so that certain models' sensory gains are higher than those of others. The wide disparities observed necessitate further refinement and continued assessment of these techniques to enhance optimum sensory reconstitution and overall patient satisfaction levels. These results have great implications for therapeutic choices as well as patient management in reconstructive surgery, particularly highlighting the capacity of these flaps to regenerate sensation.

A revision to PACE that reflects sensibility by including “S” has been proposed by this paper. By making an amendment, it acknowledges that non-innervated flaps can regain sensation after the reconstructive procedures with KDPIF.

Additionally, it draws attention to the necessity of a comprehensive evaluation of postreconstruction experiences by patients during the rehabilitation phase. These findings establish a critical base for further inquiry into how non-innervated flaps recover sensation. Such studies have the potential to be groundbreaking in terms of future developments in reconstructive surgery, leading to better sensory outcomes.

While body morphology may influence flap tension, healing, and overall outcomes, this study did not include a specific analysis of patient body habitus or its impact. Future prospective studies should investigate how individual anatomical variations affect the success and optimization of Keystone flap design.

### Conclusion

In conclusion, our study provides strong support for the wider use of KDPIF in reconstructive surgery. Its adaptability, cost-effectiveness, and reliable success rates were affirmed through a thorough evaluation with PACES. The KDPIF is a useful tool for reconstructive surgeons; it not only improves patient outcomes but also helps ensure sustainable health care system development. Patient satisfaction improvement potential, optimization of resource allocation, as well as favorable functional outcomes, proved that this approach can be utilized to further improve reconstructive surgery practice.
